# Predictors and perinatal outcomes of pre-labor rupture of membrane among pregnant women admitted to Hiwot Fana Comprehensive Specialized University Hospital, Eastern Ethiopia: a retrospective study

**DOI:** 10.3389/fmed.2023.1269024

**Published:** 2024-01-23

**Authors:** Meseret Wolde, Teshale Mulatu, Gemechu Alemayehu, Afework Alemayehu, Nega Assefa

**Affiliations:** ^1^Hiwot Fana Comprehensive Specialized University Hospital, College of Health and Medical Science, Haramaya University, Harar, Ethiopia; ^2^School of Nursing and Midwifery, College of Health and Medical Science, Haramaya University, Harar, Ethiopia; ^3^Department of Midwifery, College of Medicine and Health Sciences, Wachemo University, Hossana, Ethiopia

**Keywords:** predictors, pre-labor rupture of membrane, perinatal outcome, pregnant women, Eastern Ethiopia

## Abstract

**Background:**

Pre-labor rupture of membrane (PROM) refers to a membrane rupture that occurs after the 28th week of pregnancy but before the start of labor. If not appropriately managed, it poses a significant risk to the health of the mother and fetus. However, information on the magnitude of PROM, contributing factors, and its perinatal outcomes was limited in Eastern Ethiopia. This study assessed the prevalence, predictors, and perinatal outcomes of PROM among pregnant women admitted to Hiwot Fana Comprehensive Specialized University Hospital (HFCSUH) in Eastern Ethiopia so as to guide specific preventive measures.

**Methods:**

A hospital-based retrospective cross-sectional study was carried out from May 15 to June 14, 2022, and data were gathered by reviewing the chart records of 424 pregnant women who were admitted to maternity and labor wards in the previous two years, from January 1, 2019 to December 31, 2020. Records were chosen using a simple random sampling method. Mother’s socio-demographic traits, previous obstetric and gynecologic history, current pregnancy history, habit-related history (khat chewing), ultrasound findings, laboratory investigations, mode of delivery, maternal and perinatal outcomes were extracted from the maternal charts. Bi-variable and multivariable logistic regression analyses were performed to identify predictors of pre-labor membrane rupture. The association between the explanatory and outcome variables was expressed using an adjusted odds ratio with a 95% confidence interval.

**Results:**

The prevalence of pre-labor membrane rupture was 16.27% with 95% CI: (13.05–20.11). Among 69 women who experienced pre-labor rupture of membrane, 50 (72.5%) of them had adverse perinatal outcomes. Of all 69 neonates 17 (24.64%) were delivered with low birth weight and 20 (29%) of them were born preterm. The overall perinatal mortality rate was 10.1% or 101 per 1,000 live births. History of abortion [AOR = 2.61; 95% CI (1.09, 6.24)], urinary tract infection [AOR = 2.59; 95% CI (1.23, 5.42)], antepartum hemorrhage [AOR = 3.35; 95% CI (1.38, 8.13)], and khat chewing (a leafy plant which contains psychoactive chemical) in the current pregnancy [AOR = 2.63; 95% CI (1.49, 4.63)] were all significantly associated with pre-labor rupture of membrane.

**Conclusion:**

In this study, the magnitude of pre-labor membrane rupture was relatively high compared to the global rate. Prenatal risk identification and early detection of complications among mothers with a history of abortion, antepartum hemorrhage, urinary tract infection, and counseling on the effects of khat chewing during pregnancy are crucial to reduce the likelihood of pre-labor membrane rupture and its adverse perinatal outcome.

## 1 Introduction

Pre-labor rupture of membrane (PROM) is rupture of membranes prior to the onset of labor (1). Rupture of membrane that occurs before 37 completed weeks of gestation is referred to as preterm PROM while term PROM refers to a rupture of membrane that occurs after 37th completed weeks of gestation (2). Approximately 70% of PROM cases occur at term (3).

The magnitude of pre-labor membrane rupture (PROM) varies slightly around the globe and affects 3 to 15% of all pregnancies (3, 4). It is relatively higher in Africa. For instance, the prevalence of PROM was 13.8% in Uganda, and it ranges from 1.4 to 23.5% in Ethiopia (5–8).

Preterm prelabor rupture of membranes (PPROM) complicates up to 3% of pregnancies and term PROM complicates approximately 8% of pregnancies (2, 3). PROM has been associated with significant maternal and perinatal complications which include a high rate of Cesarean section (C/S), chorioamnionitis, abruption-placenta, cord prolapse, respiratory distress syndrome (RDS), birth asphyxia, low APGAR score (<7/10), preterm labor, low birth weight, admission to neonatal intensive care unit (NICU) and perinatal death (6, 8–10). Pre-labor membrane rupture (PROM) is responsible for 44% of perinatal morbidity and 7% of perinatal mortality (11). About one-third of preterm births, which are the main cause of newborn death, and 13–60% of intra-amniotic infections or chorioamnionitis in pregnant women are attributed to PROM (12).

Moreover, PROM has a wide range of negative effects, including maternal and neonatal mortality and morbidity, as well as national economic loss from prescription expenses, hospital stays, lost productivity, and healthcare expenditures (11, 12).

There is no recognized or clear cause for PROM. Nevertheless, numerous studies have demonstrated that socioeconomic/demographic factors (maternal age, level of education, income), behavioral factors such as smoking and khat chewing (a leafy plant with psycho active chemical content), trauma or falls, a history of abortion, a prior history of premature rupture of membranes, a history of cesarean section, abnormal vaginal discharge (vaginal discharge following genital tract infection including STI) and antepartum hemorrhage (APH) were certain factors related with the occurrence of PROM (5, 7, 10, 13–15). In addition, maternal medical conditions, multiple pregnancies, and malposition are common obstetric factors associated with the incidence of PROM (14, 16).

Different strategies have been set to reduce the adverse perinatal outcomes of PROM. A management protocol for selected obstetric cases including PROM were developed and use of prophylactic antibiotics for prolonged PROM (greater than 12 h) and steroid administration following pre-labor rupture of membranes remote from term were being implemented at all hospital levels (17). Despite all these efforts, feto-maternal complications related to PROM are still significant health problem in Ethiopia.

Assessing the modifiable or treatable risk factors of PROM is important to design strategies and interventions to prevent its complications and optimize pregnancy outcomes. Although prior studies tried to assess the magnitude and determinants of PROM at various levels and at certain times, the perinatal outcome of PROM and its predictors were not well studied in Ethiopia, specifically in this study area. Therefore, this study is intended to assess the prevalence, predictors and perinatal outcome of pre-labor rupture of membrane among pregnant women admitted to HFCSUH in Harar town, Eastern Ethiopia.

## 2 Methods and materials

### 2.1 Study setting and period

The study was done at the Hiwot Fana Comprehensive Specialized University Hospital (HFCSUH), which is situated in Harari region, Eastern Ethiopia, at a distance of 525 kilometers from Addis Ababa, the country’s capital. HFCSUH serves as a teaching hub and referral hospital and provides health care services for more than five million people in Eastern Ethiopia. It comprises four major departments (medical, surgery, pediatrics, and gynecology-obstetrics with 33, 42, 50, and 60 beds, respectively) and six minor departments (psychiatric, dental clinic, radiology unit, dermatology, ophthalmology, and chronic follow-up clinic visits). Additionally, it offers maternal health services to 5,800 pregnant women on average each year. This study was carried out from May 15, 2022 to June 14, 2022.

### 2.2 Study design and population

A retrospective cross-sectional study was undertaken among pregnant women admitted to Hiwot Fana Comprehensive Specialized University Hospital between January 1, 2019, and December 31, 2020. Randomly selected charts of pregnant women admitted to the maternity and labor ward with a gestational age of 28 weeks and above (calculated from the last normal menstrual period or an early ultrasound report obtained from medical records) were included in the study, whereas women’s charts with missing important data and pertinent variables (maternal conditions, obstetric/gynecologic history and perinatal outcomes) were excluded.

### 2.3 Sample size and sampling technique

Epi-calc statistical software was used to calculate the sample size with the double population proportion formula, taking into account variables that have a significant association with the outcome variable at *p* < 0.05, a two-sided confidence level of 95%, a margin of error of 5%, a power of 80%, and a ratio of exposed to unexposed of 1:1. Taking the previous history of urinary tract infection (UTI) as an exposure variable (outcomes among unexposed = 14.7% and outcomes among exposed = 26.74%) (5) and by adding a 10% non-response rate, the final required sample size was 429. All medical records of mothers admitted to maternity and labor ward between January 1, 2019, and December 31, 2020 were retrieved from the hospital’s archive room. Using admission-discharge register of labor and delivery logbooks as a sampling frame, all unique medical registration numbers of pregnant women’s chart or card were selected by simple random sampling until the desired sample size was achieved.

### 2.4 Data collection tool and procedure

A structured questionnaire and abstraction checklist that were initially developed in English were used to extract the data. The data collection instrument was crafted after revising pertinent literature (6–11, 15). The checklist includes socio-demographic characteristics, previous obstetric and gynecologic factors, current pregnancy-related factors, behavioral related factors (khat chewing), and perinatal outcomes. Prior to beginning the data collection, the supervisors and data collectors received a one-day training session. The data abstraction checklist and questionnaire were pretested on 5% of the sample size (*n* = 21) at another institution. Based on the findings of the pretest, adjustments and alterations were made to the tool. The validity of the tool was assessed using content validity and reliability was measured checking internal consistencies of the items (Cronbach’s α = 0.822).

Four Bachelor of Science midwives collected the data under the supervision of two Masters of Science nurses in maternity and neonatal nursing. Medical record numbers found in the labor and delivery ward registry were used to retrieve the necessary records. After that, the patient’s card was retrieved from the hospital’s archive room. All maternal records located in the admission record format and charts of Child Health and Mortality Prevention Surveillance (CHAMPS) were scrutinized to gather relevant data regarding the mother’s socio-demographic traits, previous obstetric and gynecologic history, current pregnancy history, habit-related history (khat chewing), ultrasound findings, laboratory investigations, mode of delivery and maternal and perinatal outcomes. Every day, the acquired data were reviewed for precision and comprehensiveness.

### 2.5 Operational definitions

#### 2.5.1 Pre-labor rupture of membranes (PROM)

A rupture of membrane after 28 weeks of pregnancy (after fetal viability) and before the onset of labor (4, 5). The diagnosis of PROM was made if a woman had a history of leaking or gushing watery vaginal fluid without a uterine contraction, visualization of fluid pooling in the fornix or from the cervix with speculum examination and by using a basic pH test of vaginal fluid or ferning of dried vaginal fluid identified under microscopic evaluation refined/documented by physician/professionals (5, 17).

A perinatal outcome is an outcome that can happen from the 28th week of gestation to the first week of the postpartum period. It can be adverse (unfavorable) perinatal outcomes or favorable perinatal outcomes (8, 9).

#### 2.5.2 Adverse perinatal outcome

Presence of either of the following: preterm birth, perinatal death (stillbirth and immediate newborn death), low birth weight, birth asphyxia, respiratory distress syndrome (RDS), meconium aspiration syndrome (MAS), low APGAR score (<7/10), and admission to the NICU (neonatal intensive care unit) (6, 8, 9).

#### 2.5.3 Stillbirth

A newborn that shows no signs of life at or beyond 28 weeks of pregnancy (6, 8).

#### 2.5.4 Low birth weight

A newborn weighing less than 2,500 g at birth (9, 18).

#### 2.5.5 Preterm birth

When a baby is born alive before 37 full weeks of pregnancy but after 28 weeks of pregnancy (8, 18).

#### 2.5.6 Perinatal mortality

The sum total of stillbirths, fetal deaths in pregnancies longer than 28 weeks, and early neonatal deaths that occur within the first few weeks of life (7, 9, 10).

### 2.6 Data processing and analysis

EpiData version 3.1 was used to code, clean, and double-enter the data before they were exported to SPSS version 26 for analysis. Descriptive data analysis was computed to generate proportions, frequencies and mean. The pre-labor membrane rupture was the outcome variable, and it was coded as ‘1’ for diagnosed PROM and ‘0’ for no PROM. To determine the predictors of PROM, bi-variable and multivariable logistic regression analyses were carried out. Explanatory variables with a *p*-value of less than 0.25 in the bi-variable analysis were regarded as candidates for the multi-variable logistic regression analysis. Model fitness was evaluated using the Hosmer-Lemeshow test (0.910). An adjusted odds ratio (AOR) with 95% confidence interval was used to describe the association between the independent variables and outcome variable. A *p*-value less than 0.05 were taken as significant in the final model.

## 3 Results

### 3.1 Socio-demographic characteristics

A total of 424 charts were reviewed, resulting in a response rate of 98.8%. About two-third, (64.2%) of the women were in the 20–30 age range (24.95 ± 5.26). Most of the women, 421 (99.3%) were married, and 261 (61.6%) of them were housewives. Regarding their level of education, 196 (46.2%) of the women could not read or write, and 252 (59.4%) of them lived in a rural area (Table 1).

**Table 1:** Socio-demographic characteristics of pregnant women admitted to Hiwot Fana Comprehensive Specialized University Hospital in Harar town, Eastern Ethiopia, 2022 (*n* = 424).

Variables	Categories	Frequency (*n*)	Percent (%)
Age in years	<20	53	12.5
	20–29	272	64.2
	≥ 30	99	23.3
Marital status	Married	421	99.3
	Others*	3	0.7
Occupation	Housewife	261	61.6
	Government employee	54	12.7
	Farmer	52	12.3
	Merchant	36	8.5
	Daily labor	17	4.0
	Student	4	0.9
Educational status	Unable to read or write	196	46.2
	Able to read or write	9	2.1
	Primary education	103	24.3
	Secondary education	75	17.7
	Colleges and above	41	9.7
Residence	Urban	172	40.6
	Rural	252	59.4

*Others = single, divorced.

### 3.2 Past obstetrics and gynecologic history of participants

Of the total of 424 women, 235 (55.4%) were multigravidas. Of those, 147 (62.6%) had an optimal birth interval. In addition, 37 (8.7%) of the mothers had previously encountered an abortion, while 22 (5.2%) had a previous event of preterm birth, 36 (8.5%) had previous experience of PROM, and 40 (9.4%) had previously experienced C/S delivery (Table 2).

**Table 2:** Past obstetrics and gynecological history of pregnant women admitted to Hiwot Fana Comprehensive Specialized University Hospital in Harar town, Eastern Ethiopia, 2022 (*n* = 424).

Variables	Categories	Frequency (*n*)	Percent (%)
Gravidity	Primigravida	189	44.6
	Multigravida	235	55.4
Birth interval in months (*n* = 235)	Short (<24)	88	37.4
	Optimal (≥24)	147	62.6
History of abortion	Yes	37	8.7
	No	387	91.3
Number of abortions (*n* = 30)	One	30	81.1
	Two and more	7	18.9
preterm birth history	Yes	22	5.2
	No	402	94.8
Previous PROM	Yes	36	8.5
	No	388	91.5
previous C/S	Yes	40	9.4
	No	384	90.6

C/S, cesarean section; PROM, premature rupture of membrane.

### 3.3 Current pregnancy history and behavioral/habits related factors

Of 424 women, 71 (16.7%) were admitted before 37 weeks of pregnancy. The majority, 343 (80.9%) of women, had ANC follow-up. Almost all of the women (97.6%) carried a singleton pregnancy, and the majority, 394 (92.9%) of the fetus had a cephalic presentation. About 51 (12.0%) of the women had UTI history, pregnancy-induced hypertension 66 (15.6%); late pregnancy vaginal bleeding 31 (7.3%); and 165 (38.9%) of the women had hemoglobin levels less than 11 g/dL. Regarding the behavioral habits of the women, 157 (37.0%) of them were khat chewers during the current pregnancy (Table 3).

**Table 3:** Current pregnancy history and behavioral/habits related characteristics of pregnant women admitted to Hiwot Fana Comprehensive Specialized University Hospital in Harar town, Eastern Ethiopia, 2022 (*n* = 424).

Variables	Categories	Frequency (*n*)	Percent (%)
Gestational age at admission	<37	71	16.7
	≥37	353	83.3
Antenatal visits	Yes	343	80.9
	No	81	19.1
Number of visits (*n* = 343)	One	49	14.3
	Two	142	41.4
	Three or more	152	44.3
Pregnancy type	Singleton	414	97.6
	Multiple gestation	10	2.4
UTI in recent pregnancy	Yes	51	12.0
	No	376	88.0
Pregnancy induced hypertension	Yes	66	15.6
	No	358	84.4
Accident/Trauma	Yes	3	0.7
	No	421	99.3
Fetal presentation	Cephalic	394	92.9
	Breech	24	5.7
	Others*	6	1.4
Antepartum hemorrhage	Yes	31	7.3
	No	393	92.7
Haemoglobin level	< 11 g/dL	165	38.9
	≥11 g/dL	259	61.1
Diagnosed with PROM	Yes	69	16.3
	No	355	83.7
Khat chewing	Yes	157	37.0
	No	267	63.0

^*^Others = face, compound, shoulder presentation; UTI, urinary tract infection; PROM, premature rupture of membrane.

### 3.4 Prevalence of PROM

Of 424 pregnant women, 69 [16.27% (95% CI, 13.05–20.11)] experienced PROM, of which 29 (42.00%) were preterm PROM (occurred before 37 weeks of gestation).

### 3.5 Maternal and perinatal outcomes of PROM

**Figure 1:**
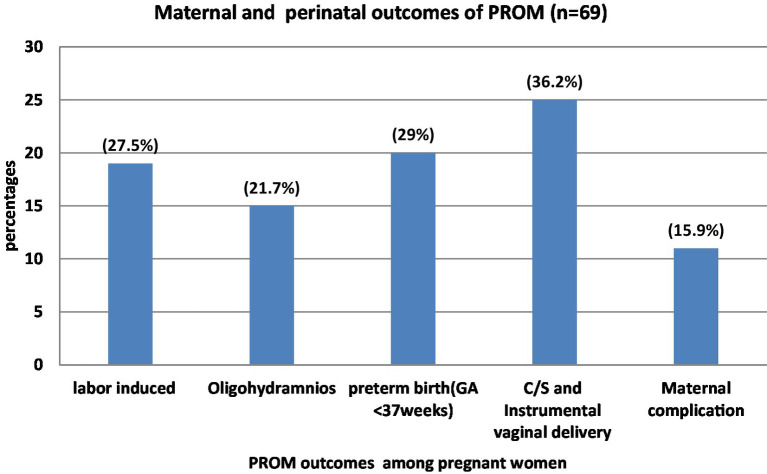
Maternal and perinatal outcomes of PROM among pregnant women admitted to Hiwot Fana comprehensive Specialized University Hospital, Harar, Eastern Ethiopia, 2022. Maternal complications include PPH, severe anemia, puerperal sepsis and retained placenta.

From a total of 69 PROM diagnoses, 50 (72.5%) of the women had adverse perinatal outcomes; 19 (27.5%) of them delivered by C/S; 15 (21.7%) experienced oligohydramnios; 19 (27.5%) underwent induction; and 20 (29%) of them delivered before 37 weeks of gestation. Eleven (15.9%) of women developed complications, of which 5 (7.2%) had postpartum hemorrhage (PPH), 2 (2.9%) had severe anemia, 3 (4.3%) had puerperal sepsis, and 1 (1.4%) had a retained placenta (Figure 1).

Concerning the neonatal outcomes, of a total of 69 neonates, 3 (4.35%) were intrauterine fetal deaths (IUFD) and 3 (4.35%) were immediate newborn deaths, and 17 (24.64%) were delivered with low birth weight. From 62 alive neonates, 19 (30.65%) have respiratory distress syndrome (RDS), 5 (8.06%) have MAS (meconium aspiration syndrome), 24 (34.75%) have a low APGAR score (<7/10) in the first 5 min, and 17 (27.42%) were referred to the neonatal intensive care unit (NICU). The overall perinatal mortality rate was 101 per 1,000 live births (10.1%) (Table 4).

**Table 4:** Neonatal outcomes among PROM diagnosed women admitted to Hiwot Fana Comprehensive Specialized University Hospital in Harar town, Eastern Ethiopia 2022 (*n* = 69).

Variable	Categories	Frequency (*n*)	Percent (%)
Neonatal condition at delivery	IUFD	3	4.35
	Still birth	1	1.45
	Immediate new born death	3	4.35
	Alive	62	89.85
Gestation at birth	Preterm (<37 weeks)	20	28.98
	Term (≥ 37 weeks)	49	71.01
Neonatal birth weight	< 1,500	2	2.90
	< 2,500	15	21.74
	≥ 2,500	52	75.36
Neonate have RDS (*n* = 62)	Yes	19	30.65
	No	43	69.35
Neonate have MAS (*n* = 62)	Yes	5	8.06
	No	57	91.94
Neonate have low APGAR in the 1st 5 min (<7/10)	Yes	24	34.78
	No	45	65.22
Neonate referred to NICU (*n* = 62)	Yes	17	27.42
	No	45	72.58

IUFD, intra uterine fetal death; RDS, respiratory distress syndrome; MAS, meconium aspiration syndrome; APGAR, appearance, pulse rate, grimace, activity, respiration; NICU, neonatal intensive care unit.

### 3.6 Predictors of PROM

In bi-variable analysis, age, abortion history, previous preterm birth, previous PROM, previous C/S, antenatal care follow-up, UTI, pregnancy-induced hypertension, antepartum hemorrhage (APH), and khat chewing were associated with PROM at *p* < 0.25. After adjusting for confounding variables, the history of abortion, UTI in the current pregnancy, antepartum hemorrhage, and khat chewing during the current pregnancy remained statistically significant in the multivariable analysis.

Those women with an abortion history had 2.6 times greater odds of PROM [AOR = 2.61; 95% CI (1.09–6.24)] when compared to women without an abortion history. Moreover, women who experienced a UTI during the current pregnancy had a 2.6-fold [AOR = 2.59; 95% CI (1.23–5.42)] increased risk of developing PROM compared to women who did not.

Additionally, those women with APH were about 3.4 times more likely [AOR = 3.35; 95% CI (1.38–8.13)] to have PROM when compared to women who did not have APH during the present pregnancy. Furthermore, the risks of developing PROM were 2.6 times greater for those women who chewed khat throughout the present pregnancy when compared to those women who did not [AOR = 2.63; 95% CI (1.49–4.63)] (Table 5).

**Table 5:** Factors associated with PROM among pregnant women admitted to Hiwot Fana Comprehensive Specialized University Hospital in Harar town, Eastern Ethiopia, 2022 (*n* = 424).

Variable	PROM		COR (95% CI)	AOR (95% CI)	*P*-value
	Yes (%)	No (%)			
Age in years					
< 20	12 (22.6)	41 (77.4)	1.80 (0.87–3.74)	1.93 (0.86–4.33)	0.109
20–29	38 (14.0)	234 (86.0)	1	1	
≥ 30	19 (19.2)	80 (80.8)	1.46 (0.80–2.68)	0.83 (0.42–1.64)	0.586
History of abortion					
Yes	12 (32.4)	25 (67.6)	2.78 (1.32–5.85)	2.61 (1.09–6.24)*	0.031
No	57 (14.7)	330 (85.3)	1	1	
History of preterm birth					
Yes	7 (31.8)	15 (68.2)	2.56 (1.00–6.53)	1.85 (0.63–5.45)	0.264
No	62 (15.4)	340 (84.6)	1	1	
History of premature rupture of membrane					
Yes	12 (33.3)	24 (66.7)	2.90 (1.38–6.13)	2.20 (0.92–5.27)	0.078
No	57 (14.7)	331 (85.3)	1	1	
History of cesarean section					
Yes	10 (25.0)	30 (75.0)	1.84 (0.85–3.96)	1.33 (0.54–3.25)	0.536
No	59 (15.4)	325 (84.6)	1	1	
Antenatal care follow up					
Yes	50 (14.6)	293 (85.4)	1	1	
No	19 (23.5)	62 (76.5)	1.79 (0.99–3.26)	1.61 (0.83–3.09)	0.157
Urinary tract infection in current pregnancy					
Yes	16 (31.4)	35 (68.6)	2.76 (1.43–5.34)	2.59 (1.23–5.42)*	0.012
No	53 (14.2)	320 (85.8)	1	1	
Pregnancy induced hypertension					
Yes	15 (22.7)	51 (77.3)	1.66 (0.87–3.15)	1.81 (0.89–3.66)	0.101
No	54 (15.1)	304 (84.9)	1	1	
Antepartum hemorrhage					
Yes	11 (35.5)	20 (64.5)	3.18 (1.45–6.98)	3.35 (1.38–8.13)*	0.008
No	58 (14.8)	335 (84.2)	1	1	
Khat chewing					
Yes	41 (26.1)	116 (73.9)	3.02 (1.78–5.12)	2.63 (1.49–4.63)*	0.001
No	28 (10.5)	239 (89.5)	1	1	

*Significant at *p* < 0.05. 1 = reference; COR, crude odd ratio; AOR, adjusted odds ratio; CI, confidence interval.

## 4 Discussion

Pre-labor rupture of membrane (PROM) is one of the significant obstetric problems that lead to morbidity and death among women and their newborns. This study assessed the prevalence, predictors, and perinatal outcomes of pre-labor membrane rupture among pregnant women admitted to Hiwot Fana Comprehensive Specialized University Hospital in Harar town, Eastern Ethiopia.

In this study, the overall prevalence of PROM was 16.3% (95% CI, 13.05–20.11). This study finding is consistent with the findings of studies conducted in Debre Tabor (13.7%) (5), Jimma (14.6%) (8), Uganda (13.8%) (19) and China (19.53%) (20). However, this result was higher than those of studies conducted in Brazil (3.1%) (13) and Tikur Anbessa Hospital in Ethiopia (1.4%) (6). The higher prevalence of PROM in our study could be attributed to inclusion criteria, as our study included all PROM diagnoses, whereas other studies only included preterm PROM. Furthermore, because the study site is a referral hospital, more severe and complicated cases of PROM were referred to and admitted to this hospital, thus increasing the prevalence of PROM.

This finding, however, was lower than those of the studies conducted in India (27.9%) (9), and Harar (23.5%), Ethiopia (7). This disparity could be attributed to the high sample size and inclusion of more than one facility, whereas our study was conducted only at one institution.

In this study, the perinatal mortality rate was 101/1000 live births (10.1%). This finding was consistent with that of Tikur Anbessa specialized teaching hospital (10.7%) (6), but higher than the study finding from Bangladesh 7% (11). This discrepancy might be due to low ANC follow-up among our study participants, where more than 27% of the women have no ANC follow-up. Another possible explanation could be due to our study facility was a referral hospital and most of the cases were referred from remote areas after they developed complications.

This study found that women with previous experiences of abortion were more prone to suffer pre-labor rupture of membranes than women with no experience of abortion. This result is congruent with research conducted in Uganda (19), Mekele (10), and a public hospital in southern Ethiopia (21). The possible reason for this might be the abortion technique, particularly dilation and curettage, may impair cervical and uterine flexibility, resulting in uterine scarring and cervical insufficiency, which results to pre-labor membrane rupture. Another possible explanation could be due to the fact that abortion can result in cervical incompetence. During abortion, mechanical dilation results in cervical trauma and may increase the risk of cervical incompetency. This might predispose membrane to trauma and rupture through effacement and dilation of the cervix (14, 22). Furthermore, the women with unsafe abortion are at greater risk to suffer from intra-amniotic and intrapartum infections, which may result in PROM (13, 23). Preventing unsafe abortion and providing high-quality post-abortion care (PAC) can help to reduce the adverse health consequences of unsafe abortion. Screening and follow-up for a mother with an abortion history are also essential to decrease the risk of PROM and its adverse perinatal outcome.

Moreover, women having a history of antepartum hemorrhage (APH) in the present pregnancy were more likely to have pre-labor membrane rupture than their counterparts. This result was consistent with the findings of research conducted at Debretabor General Hospital (5). The possible reason for this could be the formation of thrombin as a result of decidual hemorrhage may trigger the production of inflammatory cytokines and proteases, resulting in tissue necrosis, degradation of the extracellular matrix, and membrane rupture (24).

Furthermore, this study found that women who had urinary tract infections (UTIs) during pregnancy were at greater risk to have premature membrane rupture. This is consistent with study findings in rural Uganda (19) and Debretabor (5). This link could be explained by the fact that UTIs are potential reservoirs for bacteria that enter the vagina and go up through the cervical canal to the membranes, causing localized irritation. The bacteria produce a variety of proteolytic enzymes, including collagenase and gelatinase, which can cause local membrane weakening (25). Inflammatory mediators, like prostaglandins, cytokines, and proteinases in the local tissue also have a role in disrupting fetal membrane integrity and inducing uterine contractility. The generation of matrix-degrading enzymes and TNFs (tumor necrosis factors) also play a role in occurrence of PROM processes. Then, due to localized inflammation, occult contractions with increased pressure at the internal cervical os will occur, leading to PROM (24). As a result, early detection and treatment of UTIs during pregnancy are critical to reduce the risk of PROM and associated consequences.

This study also found that khat chewing is a strong predictor of premature membrane rupture. This is backed by another study finding in Eastern Ethiopia (7). Evidences showed that khat chewing practices have a negative impact on periodontal oral health (26).

The relationship between periodontal disease and PROM may be due to a probable transfer of peri-pathogenic bacteria to the placenta and amniotic fluid, as well as a systemic response to this chronic inflammatory condition (27). The possibility that chewing khat cultivated with chemical pesticides may cause significant unfavorable perinatal outcomes in khat users warrants further research.

Though pregnancy induced hypertension (PIH) and history of PROM were not significantly associated with PROM in our study, it is difficult to deny their impact on the occurrence of PROM during subsequent pregnancies. Khat chewing may increase the risk of hypertension among pregnant women which in turn may result in PIH predisposing the women for APH (particularly abruption-placenta) which is associated with PROM in our study. Hence further research is required to investigate the impact of Khat chewing on PIH and how PIH leads to APH.

### 4.1 Strength and limitations of the study

The CHAMPS (Child Health and Mortality Prevention Surveillance) evaluation or admission format sheet, as well as medical record cards, were utilized in this study. Furthermore, this study included other characteristics, such as khat chewing that had not been addressed in earlier investigations. This study also attempted to evaluate adverse perinatal outcomes related to PROM, which may provide an ideal option to anticipate PROM outcomes and prevent its complications. Because this study was conducted in a single referral center, our findings may not be applicable to people visiting lower-level facilities. Furthermore, retrospective data are susceptible to information bias. Moreover, because the data are cross-sectional, it is impossible to draw conclusions about the cause-and-effect relationship between the outcome and associated variables.

## 5 Conclusion

In this study, the magnitude of PROM was relatively high in comparison to the global rate. A history of abortion, UTI, antepartum hemorrhage, and khat chewing were all found to be risk factors for PROM. Prenatal risk identification and early detection of complications among mothers with a history of abortion and antepartum hemorrhage in the current pregnancy, screening, and treatment of UTI, and raising community awareness about the risk of khat chewing are required to minimize the occurrence of PROM and its adverse perinatal outcomes.

## Data availability statement

The original contributions presented in the study are included in the article/supplementary material, further inquiries can be directed to the corresponding author.

## Ethics statement

Ethical approval was obtained from the Institutional Health Research Ethics Review Committee of Haramaya University with a reference number of IHRERC/083/2022. A formal letter of approval and support was written to HFCSUH for permission. After properly explaining the goal and benefit of the study the permission for the study was obtained from the director of the Hospital. The data were gathered without the use of names or other identifiers. All information was used solely for research reasons, and confidentiality was maintained during the study period.

## Author contributions

MW: Conceptualization, Investigation, Data curation, Formal analysis, Funding acquisition, Methodology, Project administration, Resources, Software, Writing – original draft. TM: Conceptualization, Investigation, Supervision, Validation, Visualization, Writing – review & editing. GA: Conceptualization, Formal analysis, Investigation, Writing – review & editing. AA: Conceptualization, Investigation, Supervision, Validation, Visualization, Writing – review & editing. NA: Conceptualization, Supervision, Validation, Visualization, Writing – review & editing.
